# SAAMBE: Webserver to Predict the Charge of Binding Free Energy Caused by Amino Acids Mutations

**DOI:** 10.3390/ijms17040547

**Published:** 2016-04-12

**Authors:** Marharyta Petukh, Luogeng Dai, Emil Alexov

**Affiliations:** 1 Computational Biophysics and Bioinformatics, Physics Department, Clemson University, Clemson, SC 29634, USA; mpetukh@clemson.edu (M.P.); luogend@g.clemson.edu (L.D.); 2 Department of Computer Sciences, Clemson University, Clemson, SC 29634, USA

**Keywords:** missense mutations, energy calculation, binding free energy, MM/PBSA method

## Abstract

Predicting the effect of amino acid substitutions on protein–protein affinity (typically evaluated via the change of protein binding free energy) is important for both understanding the disease-causing mechanism of missense mutations and guiding protein engineering. In addition, researchers are also interested in understanding which energy components are mostly affected by the mutation and how the mutation affects the overall structure of the corresponding protein. Here we report a webserver, the Single Amino Acid Mutation based change in Binding free Energy (SAAMBE) webserver, which addresses the demand for tools for predicting the change of protein binding free energy. SAAMBE is an easy to use webserver, which only requires that a coordinate file be inputted and the user is provided with various, but easy to navigate, options. The user specifies the mutation position, wild type residue and type of mutation to be made. The server predicts the binding free energy change, the changes of the corresponding energy components and provides the energy minimized 3D structure of the wild type and mutant proteins for download. The SAAMBE protocol performance was tested by benchmarking the predictions against over 1300 experimentally determined changes of binding free energy and a Pearson correlation coefficient of 0.62 was obtained. How the predictions can be used for discriminating disease-causing from harmless mutations is discussed. The webserver can be accessed via http://compbio.clemson.edu/saambe_webserver/.

## 1. Introduction

Practically every protein is involved in various binding processes [1], frequently with another proteins [2,3]. Altering such interactions via amino acid substitutions, naturally occurring or engineered, is expected to have significant impact on the wild type characteristics of the cell [4,5]. While such changes can, in principle, be experimentally measured, the cost and the required time are prohibitory for large-scale investigations. Because of that, the development of computational approaches is needed for large-scale modeling of the effects of amino acid substitutions on protein–protein binding [6,7,8,9,10,11,12,13] (for comparison of popular approaches see Reference [14]). The existing computational methods are typically clustered into two main groups: approaches utilizing sequence information and approaches considering structural features [7,8,15,16]. The main advantage of the approaches using sequence data is that they are fast, but the performance of such predictors strongly hinge on the training set of data. The structure-based approaches represent the other part of the spectrum. Among them the most rigorous, but computationally demanding, are the thermodynamic integration (IT) and the free energy perturbation (FEP) methods [17]. At the same time, the Molecular Mechanical Poisson-Boltzmann (Generalized Born)/Surface Accessible (MM/PB(GB)SA) approach is a method that provides the details of the modeling while requiring reasonable computational time [18,19]. In the MM/PBSA method, the binding free energy change (ΔΔΔG) is modeled as a linear combination of several potential energies, including molecular mechanics energy, and polar and non-polar components of solvation energy.

Many disease-causing mutations affect protein–protein and protein–DNA interactions [4,9,20,21,22,23,24,25]. Thus, predicting binding free energy changes caused by amino acid mutations has implications for discriminating disease-causing mutations from harmless mutations [8,16,26]. Recently, it was demonstrated that the changes of the binding free energy are correlated with the propensity of a given mutation to be disease-causing [27], especially if the predicted energy changes are normalized to the wild type binding free energy [28]. Therefore, accurate predictions of the changes of the binding free energy are needed for detecting disease-causing mutations occurring in the human genome.

In this work we report a webserver, which utilizes a previously developed methodology termed Single Amino Acid Mutation based change in Binding free Energy (SAAMBE). SSAMBE combines two approaches: sequence- and structure-based methods [18]. It utilizes the MM/PBSA method along with an additional set of statistically delivered terms from numerical investigation of the physico-chemical properties of protein complexes (the corresponding entries are provided in Tables S1 and S2). The performance was tested against more than 1300 mutations with experimentally available changes of the binding free energy taken from 43 proteins [29] and resulted in a good agreement with experimental data (Pearson correlation coefficient 0.62). It should be pointed out that SAAMBE is fast enough to allow for large-scale calculations, since the average time for modeling a mutation is less than a minute.

## 2. Results

The SAAMBE webserver is based on the SAAMBE algorithm [18], which predicts the changes of binding free energy caused by amino acid substitutions. The SAAMBE algorithm performance was previous reported [18] and it is shown that the algorithm achieves a Pearson correlation coefficient of 0.62 in a benchmark against more than 1300 experimentally-determined changes of binding free energy. The results of benchmarking are summarized in Table 1, where the correlation coefficient for specific cases is provided: (a) case 1, when a bulky residue is changed to a small one; (b) case 2, when the mutant (MT) residue is Ala, which is typically used for predicting protein “hot-spot”; and (c) case 3, the accuracy of predictions for mutations being in a particular structural region.

It can be seen that SAAMBE does not perform very well on particular types of substitutions, but overall performance is very good, achieving *R* = 0.716 if cases within two standard deviations are considered. It was demonstrated in the original work [18] that such performance is much better than the performance of existing solutions, including BeAtMuSiC [30] and FoldX [31]. However, it should be mentioned that a recent paper [14] showed that these two approaches are not among the best predictors. Instead, newly developed methods, such as mCSM-PPI [12] and those outlined in Reference [13], perform better.

**Figure 1:**
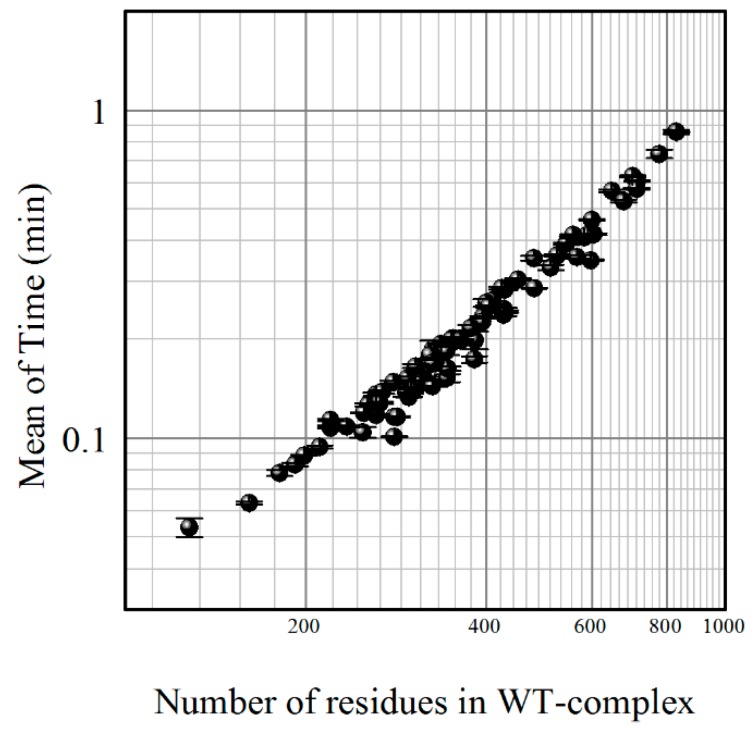
The computational time (mean value) of the Single Amino Acid Mutation based change in Binding free Energy (SAAMBE) algorithm as a function of the number of residues in the protein complex. Both axes are in log scale. WT: wild type complexes.

One of the most important characteristics of any server performance is how fast the user receives the results. The SAAMBE method produces results in 0.22 min on average when employing 16 nodes on Clemson University Palmetto Supercomputer. The time of calculations depends on the number of residues in the protein complex; Figure 1 illustrates this, and shows that, even for very large complexes of more than 800 residues, the execution time is less than a minute.

As mentioned above, the predicted binding free energy changes can be used to infer if mutations are disease-causing or harmless. Typically, this is done by setting up a particular threshold of the energy change and it is assumed that any mutation-causing effect larger than the threshold is disease-causing. Since, in this work, we do not introduce metrics for disease-association, the goal is to benchmark the performance of the SAAMBE method by setting up various thresholds for the calculated and experimental binding free energy change and to see how SAAMBE performs on matching experimentally observed large (large—above the specific cut-off) binding free energy changes. The performance is investigated using the procedure detailed in the Materials and Methods section. Several scenarios will be investigated. Below, we refer to experimentally-measured changes of the binding free energy and those calculated with SAAMBE. Further, we will apply one of the scenarios to test SAAMBE’s ability to discriminate disease-causing and polymorphic mutations.

### 2.1. Scenario 1

**Figure 2:**
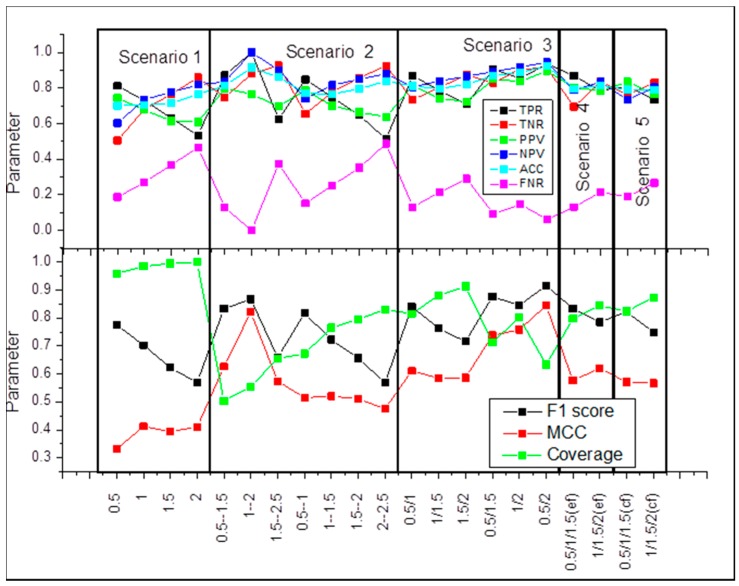
Benchmarking of statistical parameters as a function of various definitions (see Materials and Method section for details).

The cut-off for experimental and calculated ΔΔΔG is identical. In this case, the cut-off above which ΔΔΔG is considered to be large is the same for the experimental and calculated ΔΔΔG. We systematically varied the cut-off from 0.5 kcal/mol up to 2 kcal/mol in steps of 0.5 kcal/mol. The results are presented in Figure 2, scenario 1. It can be seen that the coverage is very good, reaching almost 100%, but the Matthew Correlation Coefficient (MCC) and F1 score are not impressive. At the same time, true positive ratio (TPR) and true negative ratio (TNR) are very good.

### 2.2. Scenario 2

The cut-off for experimental and calculated ΔΔΔG is identical, but there is a gap between the threshold for large and small ΔΔΔG. In terms of disease-causing and harmless mutations, this will correspond to the case for which there will be a “gray” zone of energy changes that cannot be associated, neither with disease, nor to be called harmless (similar approach was described in Reference [32] for evaluating protein stability changes). It can be seen that coverage is significantly lower as compared to scenario 1, but the MCC and F1 score are better (Figure 2, scenario 2).

### 2.3. Scenario 3

The cut-offs for experimental and calculated ΔΔΔG are different. This scenario is applicable in the case when the method over- or under-predicts the experimentally observed ΔΔΔG. The results are shown in Figure 2, scenario 3. It can be seen that this scenario achieves the best performance. The coverage reaches 0.8, without compromising MCC (MCC = 0.75) and F1 score (F1 score = 0.85) at cut-offs of 1 and 2 kcal/mol.

### 2.4. Scenario 4

The cut-offs for experimental and calculated ΔΔΔG are different, but there is a difference in defining the bottom limit (see Materials and Methods section for details). In this testing, Figure 2, scenario 4, the coverage increases, but the MCC is low.

### 2.5. Scenario 5

The cut-offs for experimental and calculated ΔΔΔG are different, but there is a difference in defining the bottom limit (see Materials and Methods section for details). As above, the coverage increases, but the MCC is low (Figure 2, scenario 5).

The above investigation indicates that, with proper definition of the cut-offs, the SAAMBE method can distinguish amino acid substitutions causing a large change of the binding free energy and to discriminate them from the substitutions causing minimal change. This paves the way for detecting disease-causing mutations, and which disease-causing effects are associated with protein–protein interactions, which will be demonstrated in the following paragraph.

### 2.6. Case Studies

To illustrate the applicability of the SAAMBE webserver to detect disease-causing mutations, and to distinguish them from polymorphic mutations, we will investigate two proteins with available clinical data. These two cases were taken from the ClinVar database [33,34] to represent proteins with available 3D structures of hetero- and homo-complexes. It should be reiterated that disease-causing mechanisms may involve altering various biophysical characteristics of the corresponding macromolecule, including stability, interactions, and dynamics [8,16,26,27,28]. Thus, if one attempts to predict disease-causing mutation based on the effect of mutation on the binding free energy, while mutation affects mostly protein stability, then the prediction will not be correct. However, here, we emphasize the ability of predicting disease-causing mutations altering macromolecular interactions.

We begin the analysis with human ribonuclease inhibitor-angiogenin complex (EC 3.1.27.-), PDB ID 1a4y [35]. Missense mutations in angiogenin are associated with amyotrophic lateral sclerosis [36,37,38]. Currently, the ClinVar database provides sixteen amino acid changes, out of which fourteen can be mapped on the available experimental structure (1a4y) of ribonuclease-angiogenin. These fourteen mutations were subjected to the SAAMBE webserver to predict their effects on the binding free energy. Chains A and B in the original Protein Data Bank (PDB) file were used. The results are shown in Table 2.

This case has a polymorphic, an unclassified, and twelve disease-causing mutations. If one considers scenario 1, described above, and selects a cut-off of 1 kcal/mol (absolute value), then SAAMBE correctly predicts five disease-causing mutations and discriminates them from the polymorphic and unclassified mutations. The disease-causing effect of the remaining ten disease-causing mutations can be associated with altering other (different from protein affinity) characteristics of native proteins.

The second case study was done to assess SAABME’s performance for mutations that are not located at a protein interface. For this purpose, we selected fructose 1,6-bisphosphate aldolase from human liver, PDB ID 1qo5 [39]. Mutations in this protein are associated with hereditary fructose intolerance [40,41,42]. The list contains eighteen mutations, out of which three are polymorphic and one is unclassified. The molecule is a homo-dimer and a mutation should be introduced on both chains. However, currently, SAAMBE is only designed to predict single mutations. This made us introduce mutations separately on chain A and B and then to sum up the effects. Independently, using the in-house SAAMBE version, we tested this approach by introducing both mutations at the same time and compared the results obtained via simple summation of the effects. Indeed, a vast majority of the cases studied here resulted in a simple cumulative effect, justifying the simple summation approach (the root mean standard deviation (RMSD) between calculated binding free energies via simple summation and introducing both mutations was 0.3 kcal/mol). The results are shown in Table 3. Thus, adopting scenario 1 and selecting a cut-off of 1 kcal/mol, allows SAABME to correctly predict harmless and unclassified mutations. In addition, SAAMBE correctly predicts five of the disease-causing mutations, despite the fact that they are not located at the protein interface. It is speculated that the remaining ten disease-causing mutations affect protein stability or another important biophysical characteristics of the monomeric protein, rather than altering protein interactions.

## 3. Discussion

The SAAMBE webserver is a simple to use tool that utilizes new algorithms for the prediction of the change of the binding free energy caused by amino acid mutations. SAAMBE predicts, not only the binding free energy changes, but reports the changes of the corresponding energy components and provides energy-minimized structures of both the wild type (WT) and the mutant type (MT). This allows the users to carry out further structural analysis of the effects of the mutations.

To assess SAAMBE’s ability to predict disease-causing mutations and to discriminate them from polymorphic mutations, we carried out analyses of two cases involving protein complexes. It was shown that SAAMBE can distinguish disease-causing and polymorphic mutations that affect protein interactions. However, since mutations may affect various biophysical characteristics of the corresponding protein, one should complement SAAMBE’s predictions with investigations of protein stability, dynamics, hydrogen bonds, and other biologically important protein features.

## 4. Materials and Methods

Definitions of mutation site locations: Here, we assign the location of mutated residues in the protein–protein complex using five distinctive categories (core (COR), support (SUP), rim (RIM), interior (INT) and surface (SUR)), as previously described [18]. This is done by calculating the relative solvent accessible surface area (SASA), which is the ratio between SASA of a residue in a protein and in water (rSASA). For example, rSASA = 1 corresponds to totally exposed residue in the protein. Thus, we calculate the SASA of the residue in the monomeric protein and term it rSASAm, and also in the complex and term it rSASAc. Finally, the term ΔrSASA refers to their mutual difference. Based on this classification, a residue is considered to be at the interface if it is assigned to COR, SUP and RIM regions; and a residue is considered to be away from the interface if it is in SUR and INT regions. The last two locations, the RIM and SUR locations, refer to residue that is exposed to the water phase in the complex. The parameters of each of the above-mentioned definitions are provided in Table 4.

Typically, the accuracy of prediction of disease-causing mutations is evaluated via the ROC parameters, adopting particular cut-offs for true and false positives. Several quantities are evaluated with Equations (1)–(9), using the relationship between four quantities: true positive (tp), true negative (tn), false positive (fp), and false negative (fn).

(1) $$\text{True Positive Rate }\left(\text{TPR},\text{ sensitivity}\right)=\frac{\text{tp}}{\text{tp}+\text{fn}}$$

(2) $$\text{False Negative Rate }\left(\text{FNR}\right)=\frac{\text{fp}}{\text{tfn}+\text{tp}}$$

(3) $$\text{True Negative Rate }\left(\text{TNR},\text{ specificity}\right)=\frac{\text{tn}}{\text{tn}+\text{fp}}$$

(4) $$\text{Positive Predictive Value }\left(\text{PPV},\text{ precision}\right)=\frac{\text{tp}}{\text{tp}+\text{fp}}$$

(5) $$\text{Negative Predictive Value }\left(\text{NPV}\right)=\frac{\text{tn}}{\text{tn}+\text{fn}}$$

(6) $$\text{Accuracy }\left(\text{ACC}\right)=\frac{\text{tp}+\text{tn}}{\text{tp}+\text{fp}+\text{tn}+\text{fn}}$$

(7) $$F1\text{ score }=\frac{2\text{tp}}{2\text{tp}+\text{fp}+\text{fn}}$$

(8) $$\text{Matthews Correlation Coefficient }\left(\text{MCC}\right)=\frac{\text{tp}\cdot \text{tn}-\text{fp}\cdot \text{fn}}{\sqrt{\left(\text{tp}+\text{fp}\right)\left(\text{tp}+\text{fn}\right)\left(\text{tn}+\text{fp}\right)\left(\text{tn}+\text{fn}\right)}}$$

(9) $$\text{Coverage }=\frac{\text{tp}+\text{tn}+\text{fp}+\text{fn}}{\text{Number of Cases in Database}}$$

Several scenarios are investigated and their definitions are provided in Table 5.

## 5. Webserver Architecture

### 5.1. Overview of SAAMBE Webserver

The design of the SAAMBE server can be described by three basic components, the client or user interface, the server and the job backend. The client interface is implemented using HTML and JavaScript). It provides the user a form to fill out various parameters for a job to be submitted to the server, as well as a button to upload the PDB file. Once the job submitted, the user will be redirected to the result page. The result page will refresh itself every 30s to get the latest results of the backend running job. The server part analyzes the parameters and writes the job and parameter files to job backend, assembles the Protein Data Bank (PDB) [43] files and starts the job remotely. The server is implemented using PHP and hosted by the Apache webserver. The job backend executes the job within a Palmetto cluster. The job handling is implemented using Python Below we describe each component of the processes in detail.

### 5.2. Client Interface

This is a web page where the user inputs all necessary information to complete a server job. The user must supply a PDB file by uploading a PDB file from their local file system. The job parameters include a partner selection part: partner 1 and 2; a mutation part: position, chain, original amino acid and mutated amino acid. Partner 1, partner 2, position, and chain are provided by the user, along with the original and mutated amino acid. A help file is provided as well, along with a particular example.

### 5.3. Server

The server acts as a middle man, it gets the PBD file and parameters from the user, puts it to the job backend, and starts the job execution in the job backend. It checks and gets the job results from the job backend upon the user’s query for the results.

### 5.4. Job Backend

Once the user has submitted a request, a job is submitted with a PBS command and executed by the Palmetto cluster. The Palmetto cluster is a supercomputer and it can give results to a user in a short time.

### 5.5. Results

If all user inputs are correct, then the computed results of their request are returned to them. The basic output for a successful job includes three files, the output txt file, the energy minimized mutant and wild type structures: MT_min PBD file and WT_min PBD file. If the job failed, then the output includes an error txt file and the user is expected to find the reason for the failure from this file. If the job is still running, the result page will also inform the user that the job is running.

## Figures and Tables

**Table 1:** Pearson correlation coefficient (*R*), the *y*-intercept and the slope of linear fit between experimental and predicted binding free energy changes. The number of cases is provided in parentheses. The results are shown for different types of mutations and summarized at the bottom for all mutations. The results of 5-fold cross validation test are shown as well.

Type of Mutaton and Mutation Site Location	Pearson Correlation Coefficient (*R*)	*y*-Intercept	Slope
Large-to-Small (173)	0.489	0.328	0.692
ALA-scanning (577)	0.488	0.268	0.695
COR, SUP (807)	0.461	0.351	0.813
RIM, SUR, INT (518)	0.478	−0.024	1.023
ALL (1326)	0.624 (0.716 ^±2SD^, 0.603 ^CV^)	1	−2.31 × 10^−5^

±2SD: within two standard deviations; CV: 5-fold cross validation test. ALA: Alanine residue; COR: core; SUP: support; RIM: rim; SUR: surface; and INT: interior type of mutation site.

**Table 2:** List of mutations and their positions, the effect provided by ClinVar, calculated ΔΔΔG and the location of mutation site within protein interface (for interfacial regions definition, see Methods). WT: wild type; MT: mutant type complexes.

WT Residue	Position	MT Residue	Effect	ΔΔΔG	Location
Q	36	L	Disease	0.00	SUP
Y	38	H	Disease	−0.42	INT
K	41	E	Disease	1.04	SUR
K	41	I	Disease	0.29	SUR
D	46	G	Disease	−1.25	SUR
S	52	N	Disease	0.00	RIM-COR
R	55	K	Disease	1.29	COR
C	63	W	Disease	0.07	INT-SUP
K	64	I	Disease	4.50	COR
I	70	V	Unclassified	0.42	INT
K	84	E	Polymorphism	0.81	SUR
P	136	L	Disease	−0.06	INT
V	137	I	Disease	0.79	SUP
H	138	R	Disease	−1.32	COR

**Table 3:** List of mutations and their positions, the effect provided by ClinVar, calculated ΔΔΔG. ΔΔΔG(A) and ΔΔΔG(B) indicate calculations made with mutation introduced in chain A and B, respectively. ΔΔΔG(A + B) is the sum of both predictions.

WT Residue	Position	MT Residue	Effect	ΔΔΔG(A)	ΔΔΔG(B)	ΔΔΔG(A + B)
I	74	T	Disease	0.50	0.29	0.79
R	134	S	Polymorphism	0.32	0.64	0.96
C	135	R	Disease	0.48	0.01	0.48
W	148	R	Unclassified	0.13	0.74	0.88
A	150	P	Disease	0.52	0.74	1.27
A	175	D	Disease	0.56	0.52	1.08
C	178	R	Disease	0.46	0.00	0.46
P	185	R	Disease	0.03	−0.01	0.03
E	207	Q	Polymorphism	0.35	0.15	0.49
V	222	F	Disease	0.32	0.61	0.93
L	229	P	Disease	0.86	0.85	1.72
L	257	P	Disease	0.80	0.69	1.49
I	268	N	Polymorphism	0.42	0.54	0.96
L	284	P	Disease	0.85	0.85	1.69
R	304	Q	Disease	0.39	0.22	0.61
R	304	W	Disease	0.27	0.24	0.52
N	335	K	Disease	-0.12	0.00	−0.12
A	338	V	Disease	0.20	0.07	0.27

**Table 4:** Parameters of the residues location types in protein-protein complex.

Location	Interface	Solvent Exposure	rSASAm	rSASAc	ΔrSASA
COR	Yes	No	>25%	<25%	>0
SUP	Yes	No	<25%	<25%	>0
RIM	Yes	Yes	any	>25%	>0
INT	No	No	any	<25%	=0
SUR	No	Yes	any	>25%	=0

**Table 5:** Conditions used for calculating the number of tp, tn, fp, and fn cases for five scenarios. Here, A corresponds to the value of calculated change of the binding free energy (ΔΔΔGcalc), while B for experimentally determined change of the binding free energy (ΔΔΔGexp).

ROC Parameters	Scenario 1	Scenario 2	Scenario 3	Scenario 4	Scenario 5
tp	A ≥ x, B ≥ x, sign(A) = sign(B)	A ≥ y, B ≥ y, sign(A) = sign(B)	A ≥ x, B ≥ y, sign(A) = sign(B)	A ≥ x, B ≥ y, sign(A) = sign(B)	A ≥ y, B ≥ x, sign(A) = sign(B)
tn	A < x, B < x	A < x, B < x	A < y, B < x	A < z, B < y	A < y, B < z
fp	A ≥ x, B < x	A ≥ y, B < x	A ≥ y, B < x	A ≥ x, B < y	A ≥ y, B < z
fn	A < x, B ≥ x	A < x, B ≥ y	A < x, B ≥ y	A < z, B ≥ y	A < y, B ≥ x
example	0.5	0.5–1	0.5/1.5	0.5/1/1.5 (ef)	0.5/1/1.5 (cf)
parameter values	x = 0.5	x = 0.5, y = 1	x = 0.5, y = 1	x = 0.5, y = 1, z = 1.5	x = 0.5, y = 1, z = 1.5

## Supplementary Materials

Supplementary materials can be found at http://www.mdpi.com/1422-0067/17/4/547/s1.
